# m^5^C regulator‐mediated methylation modification patterns and tumor microenvironment infiltration characteristics in acute myeloid leukemia

**DOI:** 10.1002/iid3.1150

**Published:** 2024-01-22

**Authors:** Qiang Wen, ShouJun Wang, Lili Hong, Siyu Shen, Yibo He, Xianfu Sheng, Xiaofen Zhuang, Shiliang Chen, Ying Wang, Haifeng Zhuang

**Affiliations:** ^1^ Department of Gynecologic Oncology Cancer Hospital of the University of Chinese Academy of Sciences (Zhejiang Cancer Hospital) Hangzhou Zhejiang China; ^2^ Department of Medicine HangZhou FuYang Hospital of Traditional Chinese Medicine Hangzhou Zhejiang China; ^3^ Department of Hematology and Transfusion The First Affiliated Hospital of Zhejiang Chinese Medical University (Zhejiang Provincial Hospital of Chinese Medicine) Hang Zhou Zhejiang China; ^4^ The First School of Clinical Medicine Zhejiang Chinese Medical University Hangzhou Zhejiang China; ^5^ Department of Clinical Lab The First Affiliated Hospital of Zhejiang Chinese Medical University (Zhejiang Provincial Hospital of Chinese Medicine) Hangzhou Zhejiang China; ^6^ Department of Clinical Research Center, Affiliated Hangzhou First People's Hospital Zhejiang University School of Medicine Hangzhou Zhejiang China

**Keywords:** acute myeloid leukemia, m^5^C methylation, prognosis, TCGA, tumor microenvironment

## Abstract

**Background:**

Recently, many studies have been conducted to examine immune response modification at epigenetic level, but the candidate effect of RNA 5‐methylcytosine (m^5^C) modification on tumor microenvironment (TME) of acute myeloid leukemia (AML) is still unknown at present.

**Methods:**

We assessed the patterns of m^5^C modification among 417 AML cases by using nine m^5^C regulators. Thereafter, we associated those identified modification patterns with TME cell infiltration features. Additionally, stepwise regression and LASSO Cox regression analyses were conducted for quantifying patterns of m^5^C modification among AML cases to establish the m^5^C‐score. Meanwhile, we validated the expression of genes in the m5C‐score model by qRT‐PCR. Finally, the present work analyzed the association between m^5^C‐score and AML clinical characteristics and prognostic outcomes.

**Results:**

In total, three different patterns of m^5^C modification (m^5^C‐clusters) were identified, and highly differentiated TME cell infiltration features were also identified. On this basis, evaluating patterns of m^5^C modification in single cancer samples was important for evaluating the immune/stromal activities in TME and for predicting prognosis. In addition, the m^5^C‐score was established, which showed a close relation with the overall survival (OS) of test and training set samples. Moreover, multivariate Cox analysis suggested that our constructed m^5^C‐score served as the independent predicting factor for the prognosis of AML (hazard ratio = 1.57, 95% confidence interval = 1.38–1.79, *p* < 1e^−5^).

**Conclusions:**

This study shows that m^5^C modification may be one of the key roles in the formation of diversity and complexity of TME. Meanwhile, assessing the patterns of m^5^C modification among individual cancer samples is of great importance, which provides insights into cell infiltration features within TME, thereby helping to develop relevant immunotherapy and predict patient prognostic outcomes.

## 1 INTRODUCTION

Acute myeloid leukemia (AML), a blood cancer with high aggressiveness, is featured by the heterogeneous molecular deformations as well as immature myeloid progenitor deposition within peripheral blood and bone marrow.^1^ In the United States, 19,940 newly diagnosed AML cases and 11,180 death cases were estimated in 2020.^2^ Currently, AML is mainly treated by chemotherapy, however, many cases develop treatment resistance or disease recurrence after the initial remission. Great efforts are made to formulate combined and/or targeted therapy for AML,^3^ but its 5‐year overall survival (OS) is as low as 30%.^4^ In this regard, elucidating the mechanism of AML progression is important for determining the suitable molecular subtype for targeted therapy (such as immunotherapy), thus improving AML prognosis.

Posttranscriptional RNA modification has a key function in different cancer types.^5^, ^6^ Genetic and epigenetic RNA histone and genetic alterations have been widely detected in cancer development, and diverse therapies are formulated accordingly, like drugs targeting hypoxia pathways or histone deacetylase inhibitors.^7^, ^8^ In a living body, more than 150 RNA modifications will experience modification into the third epigenetics layer, such as N1‐methyladenosine (m^1^A), 5‐methylcytosine (m^5^C), and N6‐methyladenosine (m^6^A).^9^, ^10^

m^5^C modification is a kind of reversible posttranscriptional RNA modification, which has a key role in modulating mRNA export and translation, stabilization localization, and alternative splicing (AS).^11^ m^5^C modification of mRNA is widely analyzed, and it has an influence on mRNAs, rRNAs, and tRNAs.^12^ m^5^C methylation is associated with diverse regulators, like m^5^C “readers”, methyltransferases, and demethylases.^13^ Among them, methyltransferase “writer” complex promotes m^5^C modification, while the diverse “reader” proteins identify and combine with methylated mRNAs, and “eraser” protein abolishes m^5^C modification by degrading the written methylation. Typically, m^5^C modification‐related adenosine demethylases, methyltransferases, and RNA‐binding proteins are referred to as m^5^C “writers” (NSUN1‐7, DNMT1‐2, and DNMT3A‐3B), m^5^C “erasers” (TET2), and m^5^C “readers” (ALYREF included) separately.^14^ m^5^C modification is increasingly identified to have an important function in different key pathophysiological events, like abnormal cell growth, apoptosis, abnormal immunomodulation, malignant transformation of tumor, developmental defects, and reduced self‐renewal capacity.^15^, ^16^, ^17^ But the representative gene signatures and m^5^C‐related regulators' significance in the diagnosis and prediction of AML prognosis remains unclear.

The therapeutic effect of immunotherapy based on immune checkpoint inhibitors (ICIs, like ICB, CTLA‐4, and PD‐1/L1) is well‐recognized in certain cases with long‐lasting responses.^18^ But many cases just gain little or even benefit from immunotherapy. Generally speaking, tumor occurrence is the process that involves several events related to genetic and epigenetic tumor cell alterations. Nonetheless, numerous works have reported tumor microenvironment (TME)'s important role in tumor occurrence. Besides, TME is also associated with tumor cell development and survival.^19^, ^20^ TME is quite complicated and involves stromal cells, including macrophages and cancer‐associated fibroblasts (CAFs), apart from tumor cells.^21^ Besides, some cells recruited at the distant site also exist, like new vessels, bone marrow‐derived cells (BMDCs, which include hematopoietic and endothelial progenitor cells), infiltrating immune cells (lymphocytes and myeloid cells), and secretory factors (like growth factors, cytokines, and chemokines).^22^, ^23^ Cancer cells stimulate biological behavioral changes through direct or indirect interaction with additional TME constituents, such as induction of proliferation and angiogenesis, inhibition of apoptosis, induction of immune resistance, and prevention of hypoxia. An increasing number of articles have suggested the complexity and diversity of TME; besides, TME has an essential part in immune evasion, tumor development, and immunotherapy response.^24^, ^25^ In this regard, it is of great significance to conduct a comprehensive analysis of the TME landscape diversity and complexity to identify diverse cancer immune phenotypes, and guide and predict the immunotherapy response. More importantly, this helps to identify more candidate biomarkers for determining immunotherapy responses in the patients and developing more therapeutic targets. This theory also exists in leukemia, which often occurs in the spleen, brain, testis, lymph node, and other potential infiltration. The microenvironment of these infiltrating sites has undergone adaptive changes such as bone marrow‐derived cell recruitment, increased vascular permeability, and chronic inflammation before metastasis, so as to provide an appropriate living environment for circulating leukemia cells, but the specific mechanism is not elaboration.

**Figure 1:**
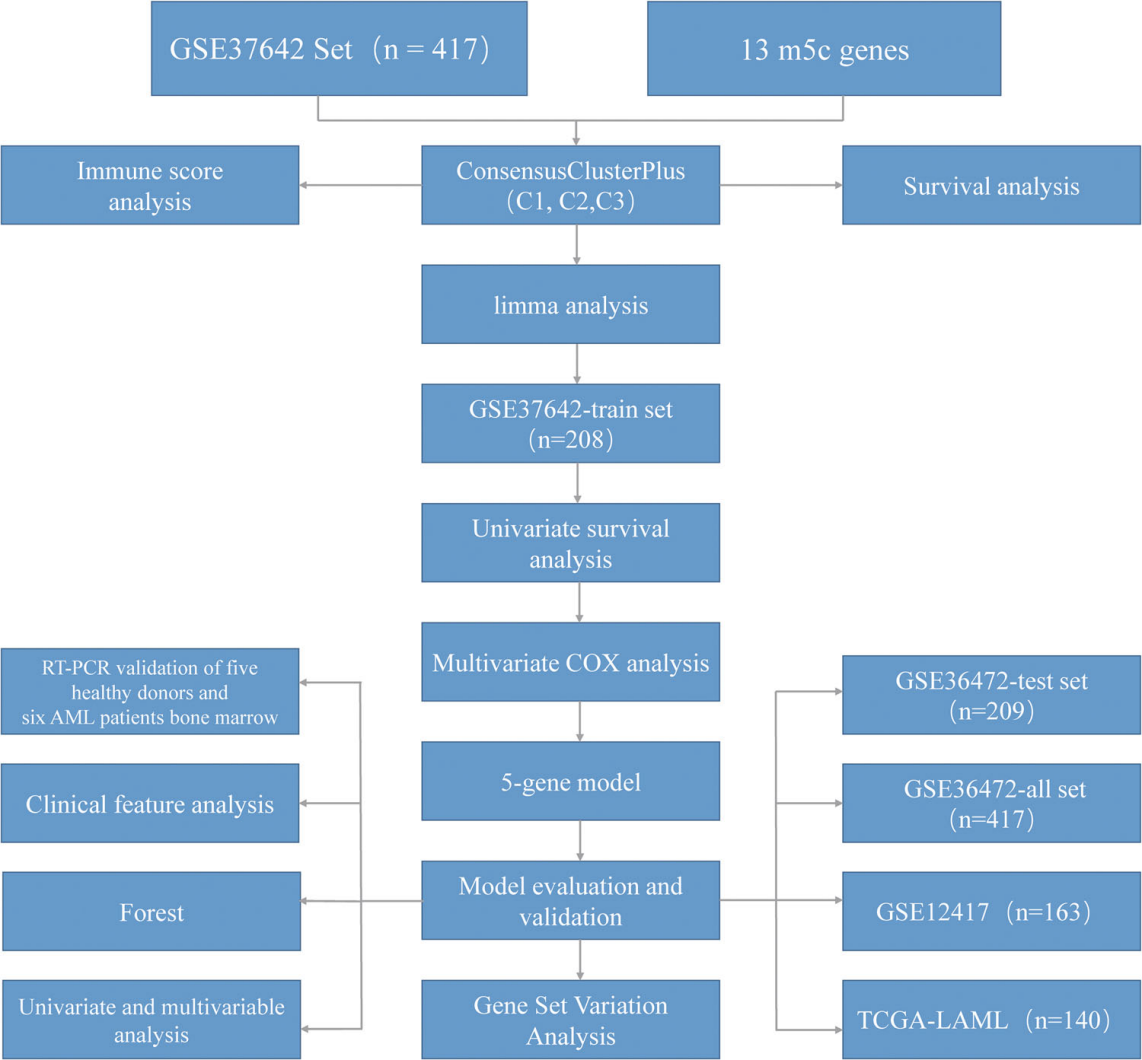
Flow chart of the present work.

Some recent articles have reported the association of tumor‐infiltrating immune cells (TIICs) within TME with m^5^C modification, which can not be interpreted by RNA degradation mechanism.^26^ However, the above studies emphasize the overall 5‐hydroxymethylcytosine (5hmC) levels and cell types because of limitations in techniques, and antitumor activity is assessed by using diverse tumor suppressors. Consequently, the comprehensive recognition of cell infiltration characteristics in TME by regulating some m5C regulators is important for better understanding TME immunomodulation. The present work (Figure 1) combined genome data from 417 GEO‐AML cases (GSE37642) for completely evaluating m^5^C modification patterns and the relations between TME cell infiltration features and clinical features. Further, the present work established a scoring system to analyzing m^5^C modification patterns for individual patients.

## 2 METHODS

### 2.1 Sources and processing of AML data

We obtained clinical data (excluding cases with no available prognosis information) and expression profiling patterns of samples in GSE12417 (*n* = 163)^27^ and GSE37642 (*n* = 417)^28^ datasets in the GEO database. Moreover, we also acquired clinical, SNP, and RNA‐seq data of 352 AML cases from TCGA database (https://cancergenome.nih.gov/) according to the following criteria, (a) enough follow‐up information; together with (b) enough gene levels in AML. Clinical data of GSE12417 and GSE37642 samples conforming to RNA‐seq data with enough expression patterns were obtained when the chip probe was mapped to gene name by adopting probe annotation file. Meanwhile, we matched the clinical data of TCGA‐derived AML samples with RNA‐seq data. We also obtained gene expression patterns after ENSG was matched with GeneSymbol.

### 2.2 13 m^5^C regulator consensus clustering

In total, we identified 13 regulators in GSE37642 cohort and adopted then in identifying different patterns of m^5^C modification regulated via m^5^C regulators. Of those 417 AML patients (GSE37642 data set), nine genes were significantly associated with disease prognosis, except for ALYREF, TET2, NSUN2, and NSUN4. Moreover, we conducted consensus clustering for identifying different patterns of m^5^C modification based on the levels of nine m^5^C regulators through 100 iterations using ConsensusClusterPlus package,^29^ so as to ensure stable classification.

### 2.3 Gene set variation analysis (GSVA) and functional annotation

For investigating diverse pathways in high versus low‐m^5^c‐score groups, we utilized the “GSVA” R package.^30^ GSVA is a nonparametric and unsupervised approach to estimate alterations of pathways among the expression data set samples. We obtained gene sets of “c2.cp.kegg.v7.0.symbols” from MSigDB database for GSVA, and adjusted *p* < .05 was indicative of statistical significance. At the same time, we conducted functional annotation by WebGestalt R package^31^ upon the FDR < 0.05 threshold.

### 2.4 Prediction of TME cell infiltration

We obtained gene signatures for 28 TIICs in the TISIDB database.^32^ Meanwhile, specific TIIC infiltration ratios of all samples were determined through a single sample Gene Set Enrichment Analysis (ssGSEA) algorithm using “GSVA” function in the R package. Microenvironment Cell Populations‐counter (MCP‐counter)^33^ can also be used to calculate the absolute abundances of different cells, including two stromal and eight immune cell types, namely, T cells, CD8^+^ T cells, NK cells, cytotoxic lymphocytes (CTLs), B lineage, myeloid DCs, monocytic lineage, endothelial cells, neutrophils, and fibroblasts.

### 2.5 Differentially expressed genes (DEGs) detected among diverse patterns of m^5^C modification

For examining genes related to m^5^C, we divided patients into three different m^5^C modification patterns according to expressions of nine m^5^C regulators. In this study, DEGs were identified among various modification patterns by empirical Bayesian method with limma R package upon the |log_2_FC | >1 and FDR < 0.01 thresholds.

### 2.6 m^5^C‐score establishment

For determining patterns of m^5^C modification within the individual cancer samples, we establish the m^5^C‐score system below. First, we normalized DEGs levels obtained based on diverse m^5^C‐clusters. Thereafter, we utilized the doBy sampleBy function to stratify 417 GEO‐AML cases (from GSE37642 data set) in the verification set (*n* = 209) and training set (*n* = 208) at the 0.5 sampling proportion parameter (Table 1). Later, we analyzed the training set sample prognosis for determining the representative DEGs with univariate Cox regression model. The representative genes were then acquired for later analyses. In total, we selected 18 genes upon the *p* < .01 threshold (Supporting Information S2: Table S1). Subsequently, stepwise and LASSO regression was conducted to select the top prognostic genes from training set samples. LASSO regression is usually adopted to screen variables for fitting the high‐dimensional generalized linear model. We adopted LASSO regression in the present work for constructing the penalty function, and this contributed to obtaining an optimized model that involved less variables while preventing overfitting. Additionally, glmnet package was also employed to determine the penalty parameter lambda by means of cross‐validation. Thus, the optimal lambda value related to the smallest error mean of cross‐validation was obtained. Later, the best gene group (*λ* = 0.0485) was chosen to construct the model later. Furthermore, MASS package stepAIC method was employed to conduct stepwise multivariate regression according to feature gene expression levels. Beginning from the most complex model, one single variable was eliminated every time successively to reduce Akaike information criterion (AIC) value (the lower value stood for the superior model, indicating the use of fewer parameters in the model for obtaining the sufficient degree of fitting). Then, we chose the gene sets that had optimal AIC values for the construction of m^5^c‐score model. Afterward, we determined m^5^c‐scores based on corresponding gene expression levels and classified cases in high‐ or low‐level groups. Meanwhile, the independent prognosis prediction ability of m^5^c‐score was assessed by multivariate Cox regression.

**Table 1:** Sample information of training and verification set of GSE37642 data set.

Clinical features	GSE37642	GSE12417	TCGA‐LAML
**OS**			
0	109	60	53
1	308	103	87
**FAB**			
M0	14		
M1	84		
M2	117		
M3	19		
M4	104		
M5	47		
M6	15		
M7	2		
**RUNX1‐RUNX1T1**			
Yes	23		
No	394		
**RUNX1 mutation**			
Yes	59		
No	311		
**Age**			
≤60	238		
>60	179		

Abbreviations: OS, overall survival.

### 2.7 Validation of genes in m^5^C‐score model by qRT‐PCR

Bone marrow samples were collected from five healthy donors and six AML patients from the First Affiliated Hospital of Zhejiang University of Traditional Chinese Medicine. The study was conducted with informed consent and approved by the Ethics Committee of the First Affiliated Hospital of Zhejiang University of Traditional Chinese Medicine. Mononuclear cells were isolated from the bone marrow samples by Ficoll method. Isolation of Total RNA from Mononuclear cells was carried out using RNeasy mini kit (Servicebio). For PCR amplification, the primers were applied as follows:

ITGA4, forward: 5′‐AAGGACTACATCATCAAAGACCCAA‐3′, reverse: 5′‐AAGCCAGCCTTCCACATAACATA‐3′, the expected fragments were 118 bp;

IGLL1, forward: 5′‐GGTTTCAATCCAAGCATAACTCAG‐3′, reverse: 5′‐ GTACTTGTTGTTGCTCTGTTTGGA‐3′, the expected fragments were 263 bp;

LAPTM4B, forward: 5′‐CCATTCAGGAATACATACGGCAA‐3′, reverse: 5′‐AAGGACCAAACAGGTAGGATTCACT‐3′, the expected fragments were 86 bp;

HIST1H2AE, forward: 5′‐TCGGGCAAAAGCTAAAACGC‐3′, reverse: 5′‐TAGTTGCCTTTGCGGAGGAG‐3′, the expected fragments were 87 bp;

HOPX, forward: 5′‐CACAGAGGACCAGGTGGAAATC‐3′, reverse: 5′‐CTTAAACCATTTCTGGGTCTCCT‐3′, the expected fragments were 130 bp;

GAPDH, forward: 5′‐GGAAGCTTGTCATCAATGGAAATC‐3′, reverse: 5′‐TGATGACCCTTTTGGCTCCC‐3′, the expected fragments were 168 bp;

Three replicates of each sample were performed, and GAPDH was used as a control. The relative expression levels were determined using the 2^(−△△CT)^ method. The differences in ITGA4, IGLL1, LAPTM4B, HIST1H2AE, and HOPX expression were tested by Weltch *t*' test. R (version 3.6.3) was used to create the graphs.

### 2.8 Statistical analysis

All methods were carried out in accordance with relevant guidelines and regulations. Kruskal–Wallis test and one‐way analysis of variance were adopted to compare differences among multiple groups. Based on the relation of m^5^C‐score with patient survival, we adopted survminer R package to determine thresholds for respective datasets. Further, we adopted the “surv‐cutpoint” function for dichotimizing m^5^C‐score by calculating the candidate thresholds to discover the maximal rank statistic. Then, all cases were classified as low or high m^5^C‐score group according to our determined maximal log‐rank statistics, for the sake of decreasing calculation batch effect. At the same time, Kaplan–Meier method and log‐rank test were conducted to identify the significance of difference so as to generate survival curves. At the same time, the receiver operating characteristic (ROC) curves were plotted, and the area under the curve (AUC) values were determined by pROC R package to assess the sensitivity and specificity of m^5^C‐score. *p* < .05 (two‐sided) was indicative of statistical significance. Data were analyzed by R 3.6.1 software.

## 3 RESULTS

### 3.1 The m^5^C modification patterns of AML mediated by 9 regulators

**Figure 2:**
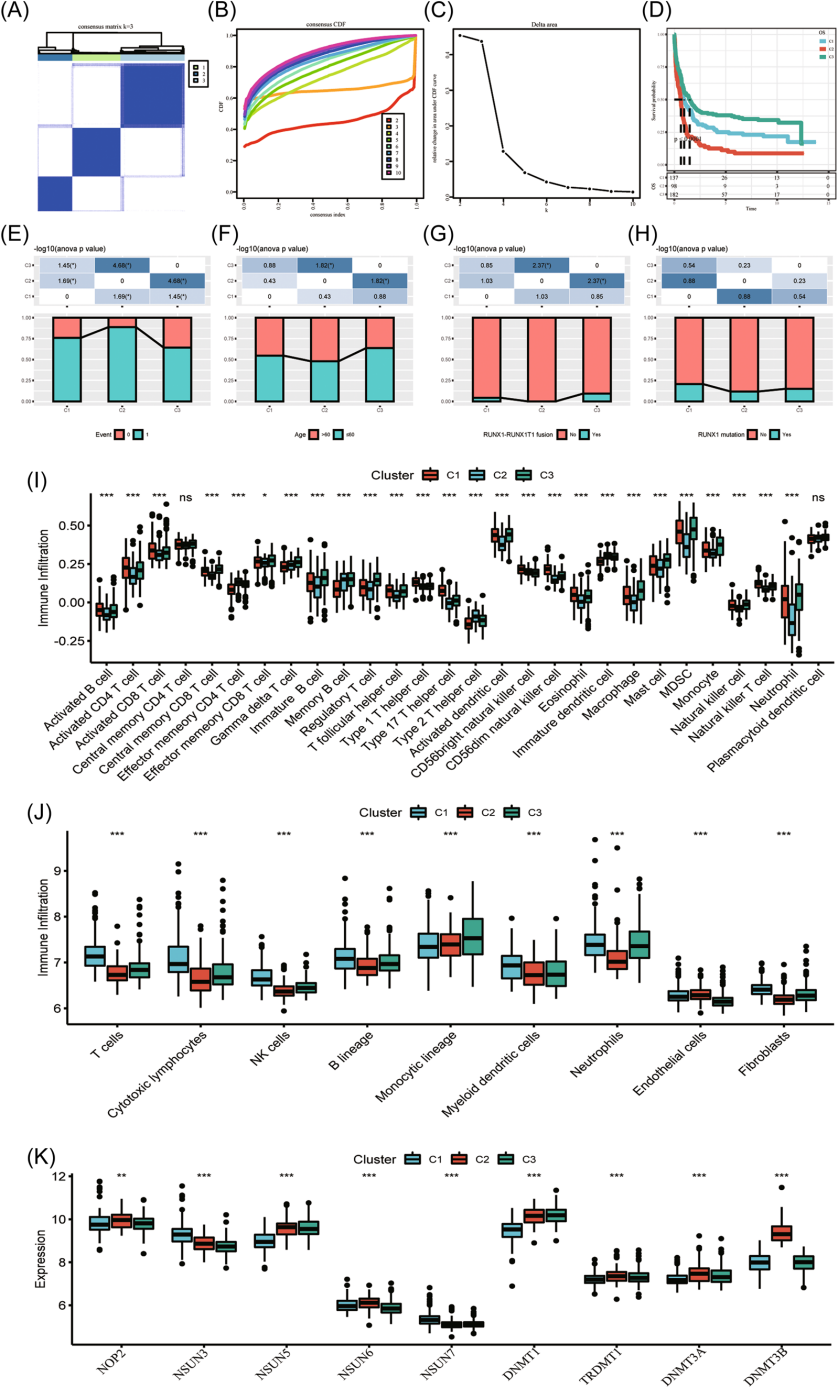
Patterns of m^5^C modification mediated via nine regulators (A–C) Different patterns of m^5^C modification were identified by consensus clustering (D) Survival analysis of diverse subtypes. Clinical feature distribution (survival rate (E), age (F), RUNX1–RUNX1T1 fusion (G), and RUNX1 mutation (H)) of samples in three m^5^C‐clusters. TIIC features across different m^5^C modification patterns (I) Enrichment scores of 28 immune cells‐related signatures in the m^5^C modification patterns (J) Infiltrating levels of nine TME cells in the m^5^C modification patterns (K) Levels of nine m^5^C regulators of the m^5^C modification patterns. m^5^C, 5‐methylcytosine; TIIC, tumor‐infiltrating immune cell; TME, tumor microenvironment.

Among the 13 m^5^C regulators, nine (DNMT3A‐3B, DNMT1‐2, NSUN1‐7, DNMT3A, and NSUN5) were significantly related to prognosis of 417 AML samples derived from GEO database (GSE37642 data set). Later, three different patterns of m^5^C modification were obtained based on the levels of nine m^5^C regulators (Figure 2A–C), also called m^5^C‐cluster 1–3. As shown in Figure 2K, the levels of nine m^5^C regulators showed significant differences among samples from three diverse subtypes. The prognostic outcomes of three m^5^C modification patterns were examined, which indicated that m^5^C‐cluster three subtype exhibited OS advantage, whereas m^5^C‐cluster 2 subtype showed OS disadvantage (Figure 2D).

### 3.2 Clinical and TIIC features in different patterns of m^5^C modification

First, distributions of diverse clinical features were compared among those 3 m^5^C‐clusters. As a result, differences in age, RUNX1–RUNX1T1 fusion, and OS rate were significant among three subtypes, whereas differences in RUNX1 mutation showed no significance (Figure 2E–H). The m^5^C‐cluster two subtype samples that had the most dismal prognostic outcome were associated with more advanced age, as well as lower RUNX1–RUNX1T1 fusion proportion and OS rate.

In addition, this study adopted ssGSEA to characterize those immune cell components among the three m^5^C‐clusters through scoring 28 signatures identified in GEO‐AML samples (GSE37642). According to Figure 2I, there were significant differences in the enrichment scores of 28 signatures among the samples, conforming to the different prognostic outcomes across the three subtypes. For example, B cells, NK cells, and activated T cells had remarkably increased scores in m^5^C‐cluster 1 in comparison with m^5^C‐cluster 2 and 3, whereas T regulatory cells, MDSCs and monocytes had increased scores in m^5^C‐cluster 3 compared with m^5^C‐cluster 1 and 2. Additionally, we also utilized MCP counter to verify the above‐mentioned results (Figure 2J), and similar results were obtained. In other words, multiple types of immune cells in TME were significantly enriched in m^5^C‐cluster 1 samples compared with samples in the other two subtypes.

### 3.3 DEGs as well as the biological behaviors across different patterns of m^5^C modification

This study adopted limma package to determine DEGs related to m^5^C phenotype upon the thresholds of |log2FC | >1 and FDR < 0.05. As a result, 48 DEGs were detected between m^5^C‐cluster 1 and cluster 3 (including three with upregulation while 45 with downregulation, Supporting Information S3: Table S2), 161 DEGs were discovered between m^5^C‐cluster 2 and cluster 1 (which included 133 with upregulation, whereas 28 with downregulation, Supporting Information S3: Table S2), while 73 DEGs were discovered between m^5^C‐cluster 3 and cluster 2 subtypes (21 with upregulation, while 52 with downregulation, and Supporting Information S3: Table S2). Moreover, we performed GO enrichment analyses on DEGs using the WebGestaltR package (Supporting Information S1: Figure S1). These genes were mostly enriched into molecular biological function related to immune inflammatory response (like neutrophil activation and leukocyte migration) (Supporting Information S4: Table S3).

### 3.4 m^5^C‐Score establishment for AML patients

**Figure 3:**
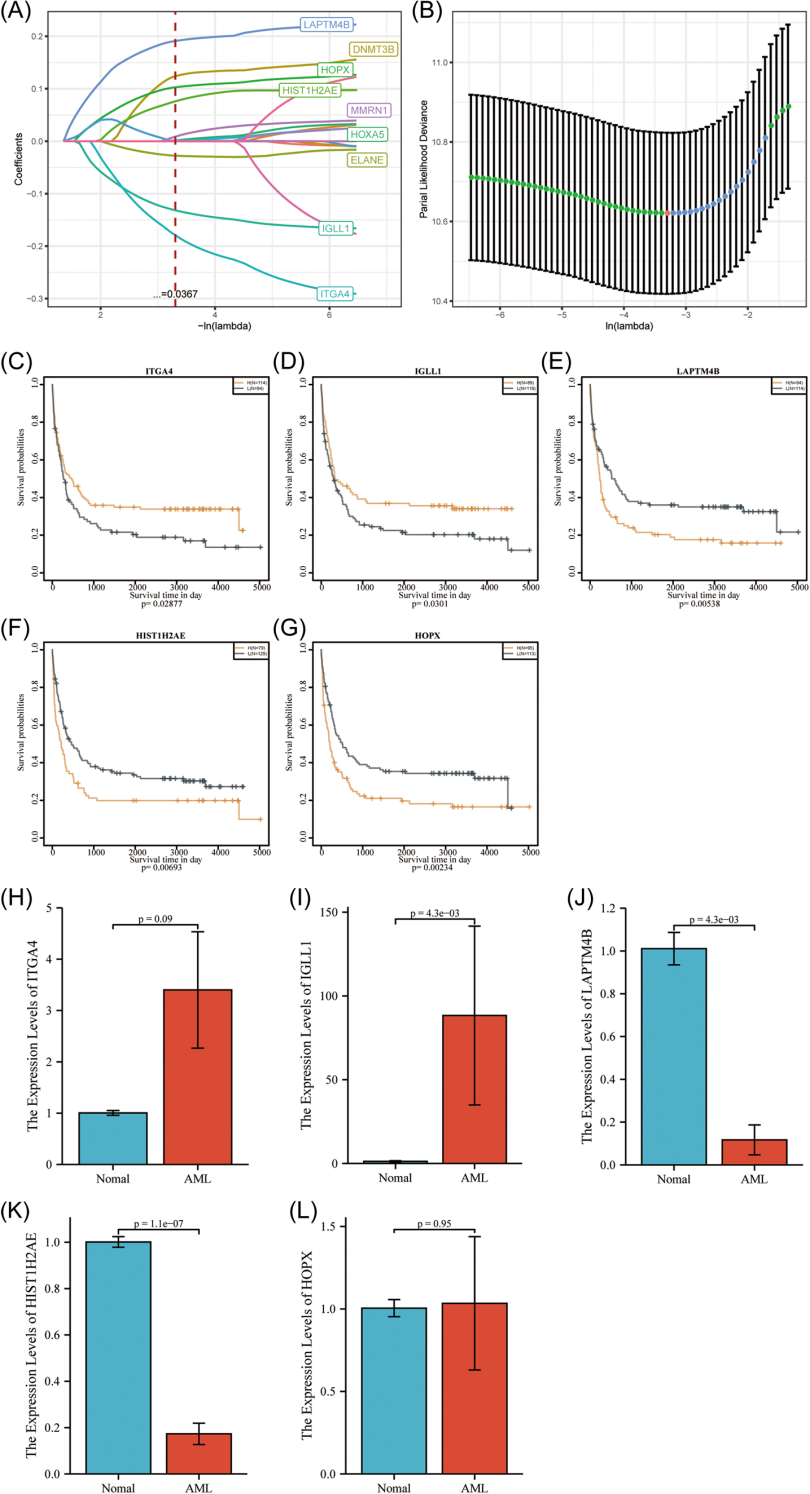
(A) The change track of each independent variable, the horizontal axis represents the log value of the independent variable lambda, and the vertical axis represents the coefficient of the independent variable. (B) Confidence intervals for each lambda. The difference in OS of cases showing high compared with low levels of those five typical prognostic genes (ITGA4 (C), IGLL1 (D), LAPTM4B (E), HIST1H2AE (F), and HOPX (G)) related to m^5^C modification patterns. Validate the m^5^C‐score model by RT‐PCR method. (H–L) Relative expression of ITGA4, IGLL, LAPTM4B, HIST1H2AE, and HOPX in bone marrows of AML patients and healthy donors. AML, acute myeloid leukemia; m^5^C, 5‐methylcytosine; OS, overall survival; RT‐PCR, real‐time polymerase chain reaction.

According to the above‐mentioned findings, m^5^C methylation modification had a key function in the formation of diverse TME landscapes as well as the regulation of AML progression or tumor immunity. However, these results were obtained from the patient cohorts, which could not precisely estimate the patterns of m^5^C methylation modification in individual cases. Since m^5^C modification was diverse and complex in individual samples, this study also established a scoring system (m^5^C‐score) using the DEGs related to phenotype, for the sake of determining m^5^C modification patterns in individual AML samples. To be specific, GEO‐AML patients (GSE37642) were first divided as training set or test set following the description in Methods. Then, survival data from samples of training set were utilized for univariate analysis on DEGs after the 3 m^5^C‐clusters were compared pairwise. We identified altogether 18 prognostic genes. Subsequently, stepwise and Lasso regression analyses were conducted for screening 5 typical prognostic genes related to m^5^C modification patterns (ITGA4, IGLL1, LAPTM4B, HIST1H2AE, and HOPX; Figure 3C–G). Based on the expressions of five genes, a m^5^C‐score was constructed for AML samples:

$$m^{5}C-\text{score}=-0.2283059\times \text{ITGA}4-0.1575680\times \text{IGLL}1+0.2686156\times \text{LAPTM}4B+0.1220958\times \text{HIST}1H2\text{AE}+0.1472148\times \text{HOPX}$$

**Figure 4:**
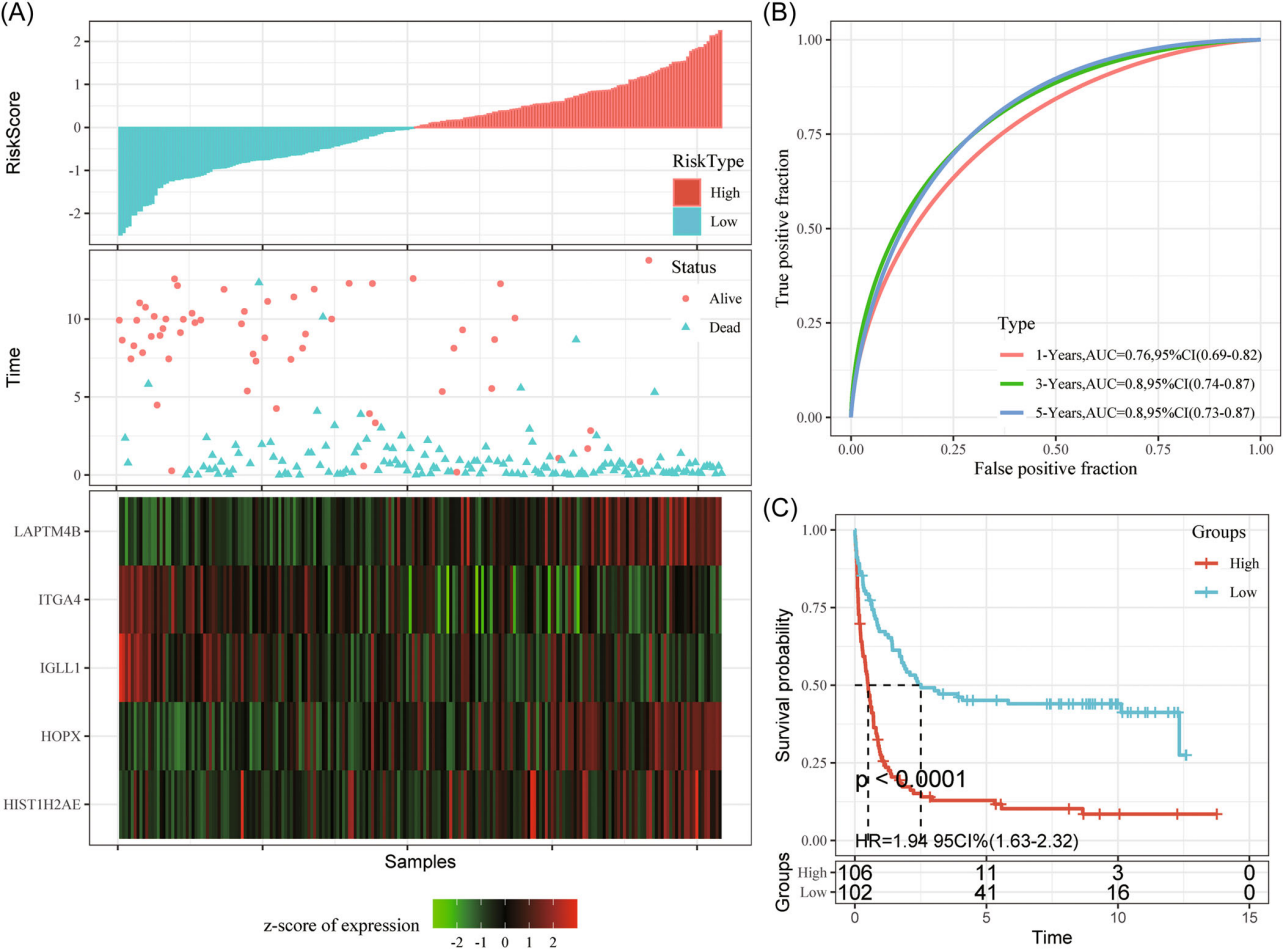
To verify the effectiveness of the m^5^C‐score model in classifying the prognosis of AML cases in the training group (A–C); gGene expression levels with OS and m5C‐score. ROC curves show the specificity and sensitivity of our established m5C‐score to the samples. Prognostic differences of samples. AML, acute myeloid leukemia; AUC, area under the curve; HR, hazard ratio; CI, confidence interval; m^5^C, 5‐methylcytosine; OS, overall survival; ROC, receiver operating characteristic.

Later, m^5^C‐scores for all training set samples were calculated and converted to z‐score. Then, we divided samples whose m^5^C‐score was >0 into the high‐level (risk) group, whereas those whose m5C‐score was <0 into the low‐level (risk) group. According to Figure 4A,C, the high‐risk group had remarkably more death samples compared with the low‐risk group, besides, samples that had high m5C‐scores were associated with dismal prognostic outcomes. As revealed by ROC analysis presented in Figure 4B, m^5^C‐score established according to five gene expression levels were highly efficient in classifying 1–5 year survival for AML samples in the training set (AUC: 0.76–0.8).

Besides, there were significant differences in clinical features (such as age, m^5^C cluster, RUNX1 mutation, and RUNX1‐RUNX1T1 fusion) between low and high m^5^C‐score groups (Figure 9G–J). Samples with low m^5^C‐scores were associated with low age, high RUNX1‐RUNX1T1 fusion proportion, and m^5^C‐cluster 3 distribution. In addition, GSVA was conducted to analyze differences in biological behaviors between low and high m^5^C‐score groups. According to Figure 9K–P, high m^5^C‐score group was mostly related to cancer‐related pathways, tight junctions, and ABC transporters, whereas the low m^5^C‐score group was mostly related to certain metabolic pathways, including glycan degradation and purine metabolism.

### 3.5 Validation of m^5^C‐score model

Five genes were selected in m^5^C‐score model (ITGA4, IGLL, LAPTM4B, HIST1H2AE, and HOPX). These genes were tested in bone marrows of healthy donors and six AML patients. The results indicated that the expression of ITG4A, and IGLL1 was significantly higher in the AML group than in the nomal group (*p* < 0.05) (Figure 3H,I). The expression of LAPTM4B and HIST1H2AE was significantly lower in AML group than in the nomal group (*p* < 0.05) (Figure 3J,K). In contrast, there was no significant difference in HOPX between the two groups (*p* > 0.05) (Figure 3L).

### 3.6 Robust performance of m^5^C‐score in the classification of prognosis for AML samples validated through internal and external datasets

**Figure 5:**
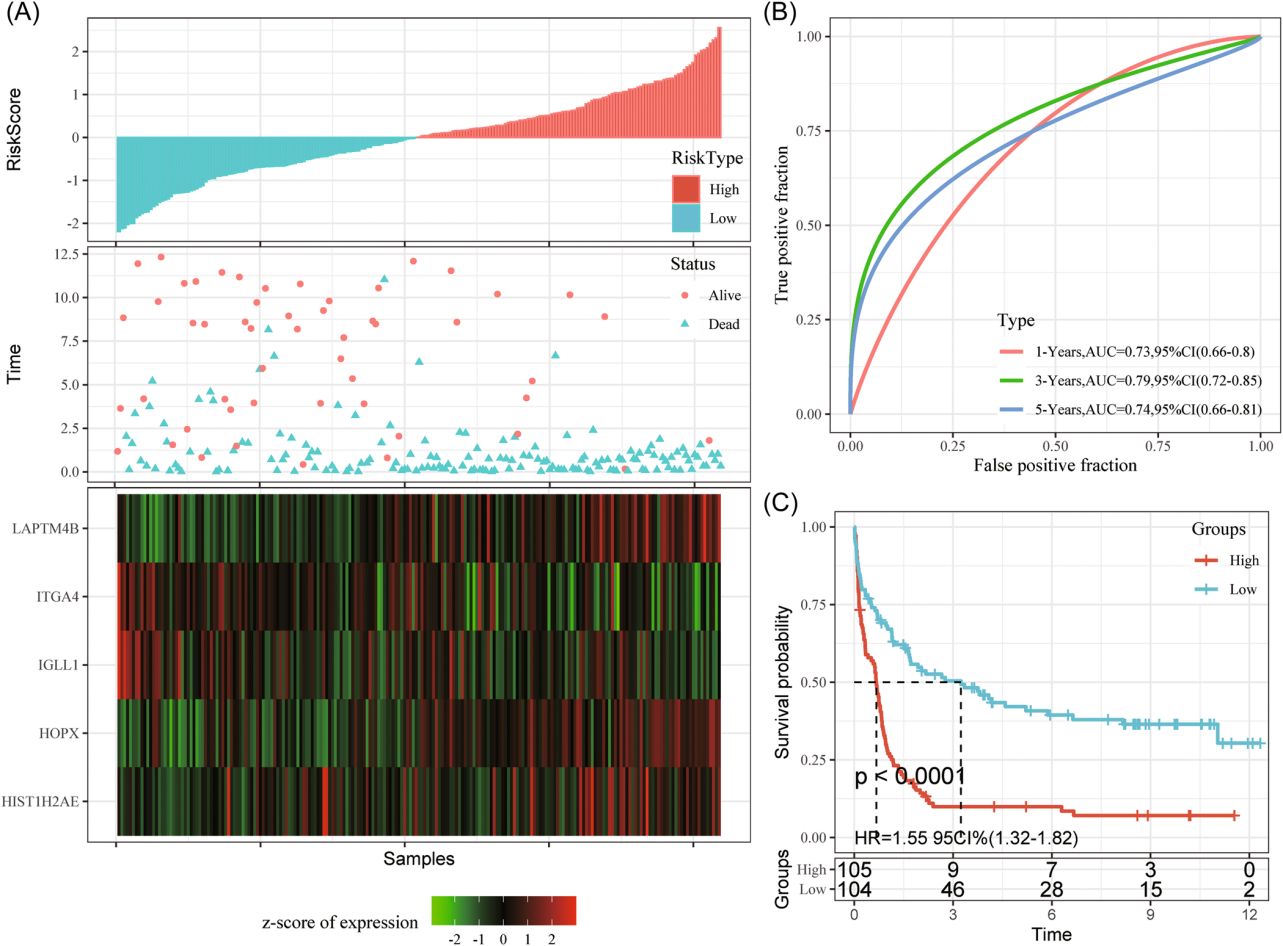
The test set of AML cases in the GSE37642 cohort (A–C); Gene expression levels with OS and m^5^C‐score. ROC curves show the specificity and sensitivity of our established m^5^C‐score to the samples. Prognostic differences of samples. AML, acute myeloid leukemia; AUC, area under the curve; CI, confidence interval; HR, hazard ratio; m^5^C, 5‐methylcytosine; OS, overall survival; ROC, receiver operating characteristic.

First, m^5^C‐scores for all samples from GSE37642 test set were calculated and converted to z‐scores. Then, we assigned samples whose m^5^C‐score was >0 as high‐risk group, whereas those whose m^5^C‐score was <0 as the low‐risk group. According to Figure 5A,C, there were remarkably more dead samples from the high‐risk group than the low‐risk group, besides, the high‐risk group had a dismal prognostic outcome. According to ROC analysis observed from Figure 5B, our m^5^C‐score established according to five gene expression levels was highly efficient in classifying 1–5 year survival for TCGA‐AML samples from the training set (AUC: 0.73–0.79).

**Figure 6:**
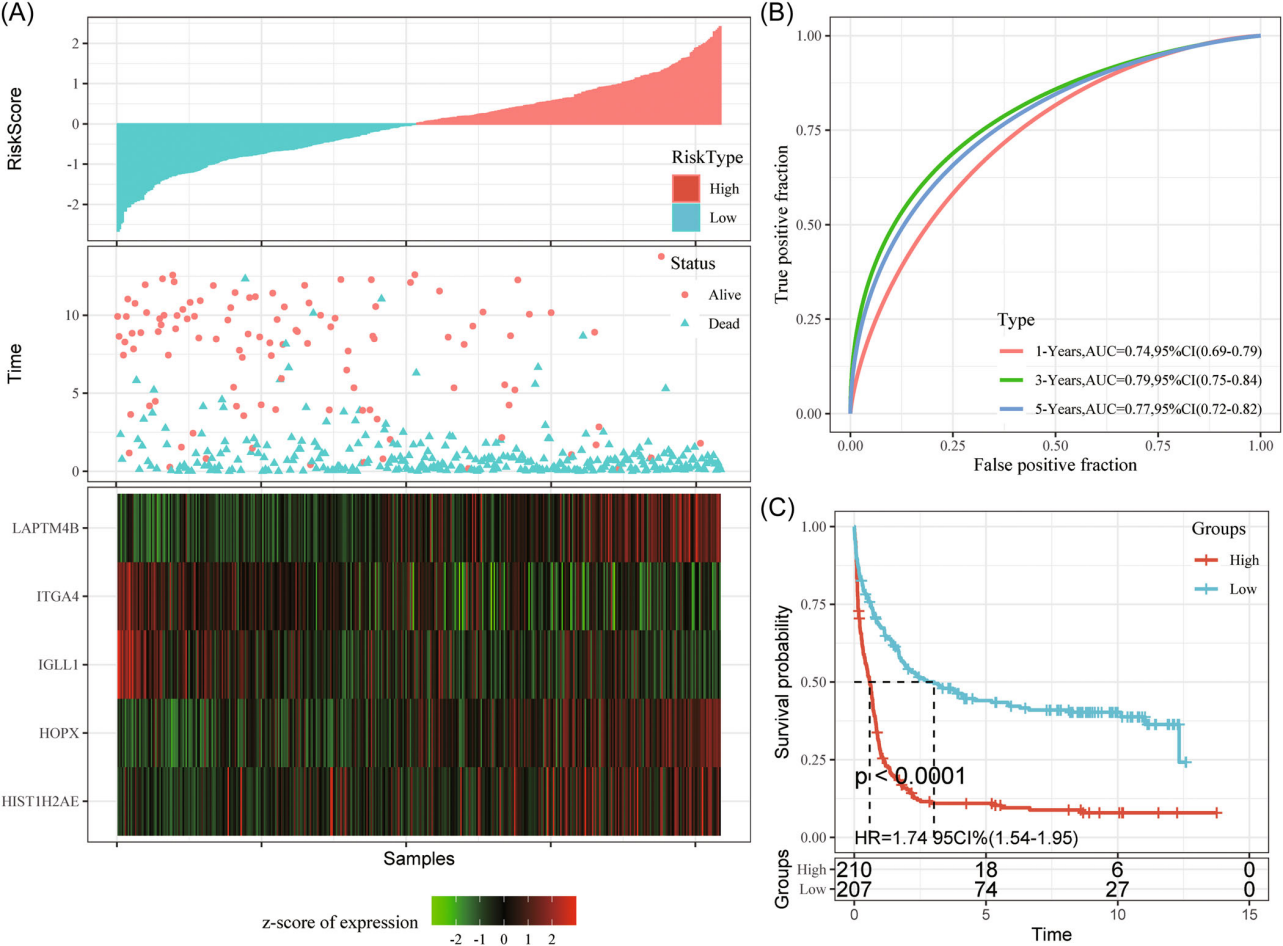
All samples in the GSE37642 cohort (A–C); gene expression levels with OS and m^5^C‐score. ROC curves show the specificity and sensitivity of our established m^5^C‐score to the samples. Prognostic differences of samples. AUC, area under the curve; CI, confidence interval; HR, hazard ratio; m^5^C, 5‐methylcytosine; OS, overall survival; ROC, receiver operating characteristic.

Subsequently, data from training set were combined with those from test set, then, the classification performance of m^5^C‐score for prognosis prediction of 417 GEO‐AML samples (GSE37642) was analyzed. As exhibited in Figure 6A,C, there were significantly more dead samples from the high‐risk group than those from the low‐risk group, with dismal prognostic outcomes of the high‐risk group. According to ROC analysis observed from Figure 6B, our m^5^C‐score established according to five gene expression levels was highly efficient in classifying 1–5 year survival for internal cohort samples (AUC: 0.74–0.79). In addition, the accuracy in predicting prognosis was enhanced as sample survival extended.

**Figure 7:**
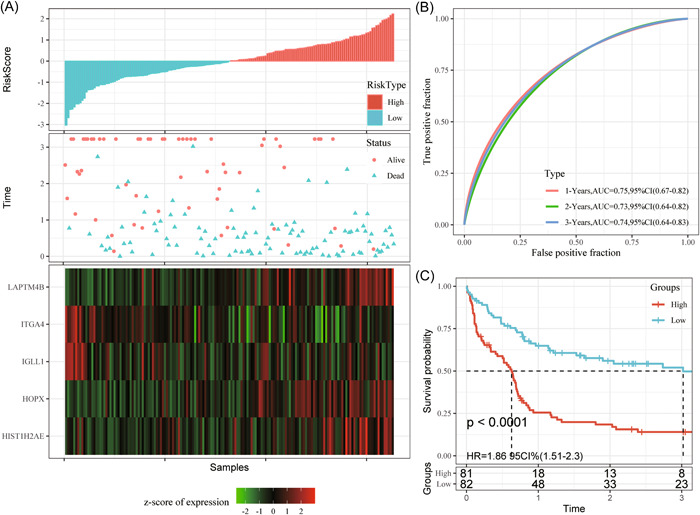
All samples in the GSE12417 cohort (A–C), gene expression levels with OS and m^5^C‐score. ROC curves show the specificity and sensitivity of our established m^5^C‐score to the samples. Prognostic differences of samples. AUC, area under the curve; CI, confidence interval; HR, hazard ratio; m^5^C, 5‐methylcytosine; OS, overall survival; ROC, receiver operating characteristic.

**Figure 8:**
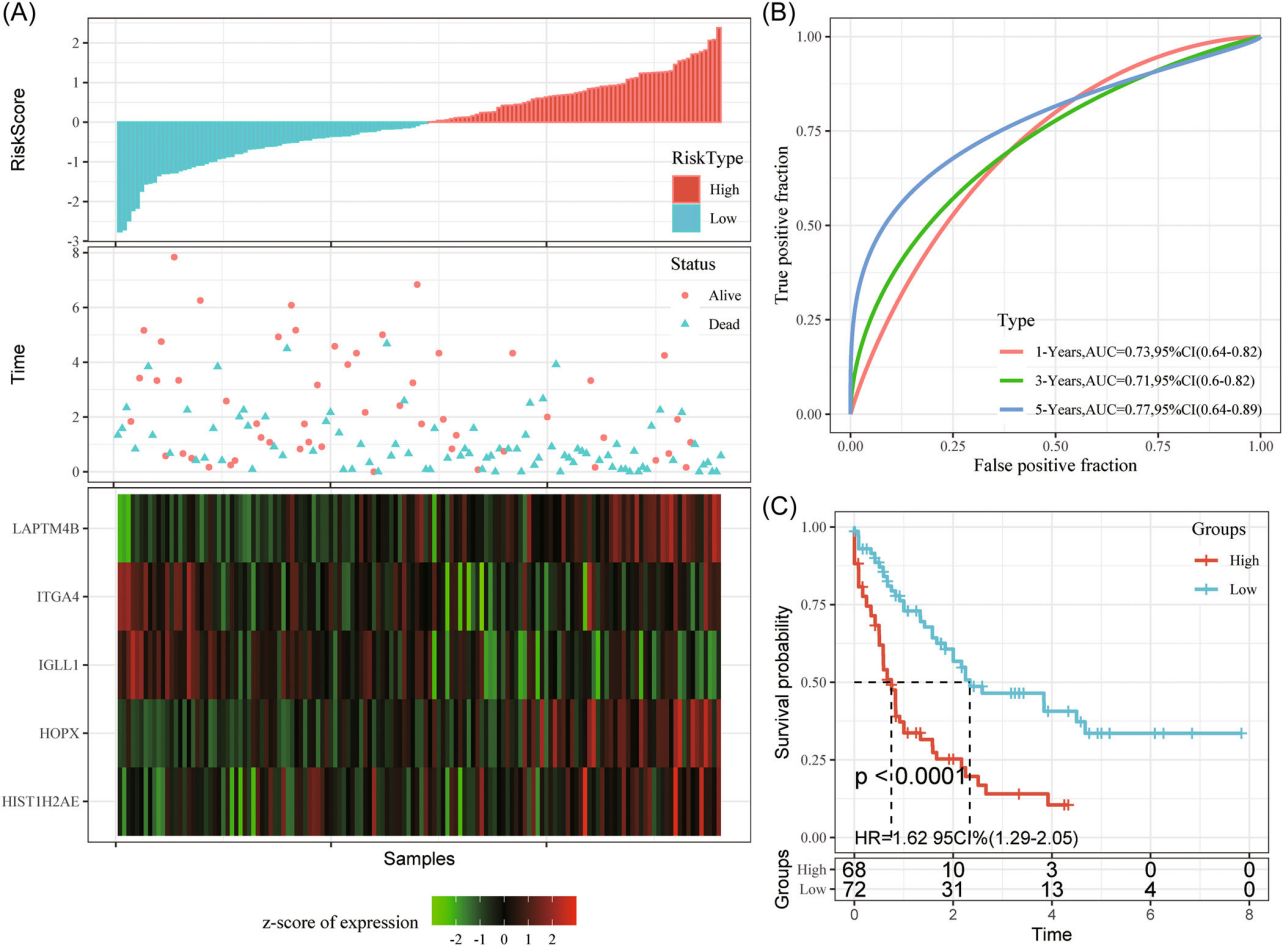
For the prognosis of TCGA‐AML cases (A–C) when classifying Robustness. Gene expression levels with OS and m^5^C‐score. ROC curves show the specificity and sensitivity of our established m^5^C‐score to the samples. Prognostic differences of samples. AML, acute myeloid leukemia; AUC, area under the curve; CI, confidence interval; HR, hazard ratio; m^5^C, 5‐methylcytosine; OS, overall survival; ROC, receiver operating characteristic.

At last, the GSE12417 (second test set) and TCGA‐AML (third test set) cohorts were obtained as the external data set, and our model was utilized for determining the m^5^C‐scores of samples. In addition, patients were classified as high‐ and low‐risk groups with the same threshold selected for training set. It was illustrated from Figures 7A–C and 8A–C that, low‐risk patients had better prognostic outcomes than high‐risk patients. ROC analysis suggested similar 1–5‐year AUC values to the training set and test set of the internal cohort. To sum up, the m^5^C‐score showed good performance in AML prognosis prediction.

### 3.7 m^5^C‐score served as the factor to independently predict the prognosis of AML

**Figure 9:**
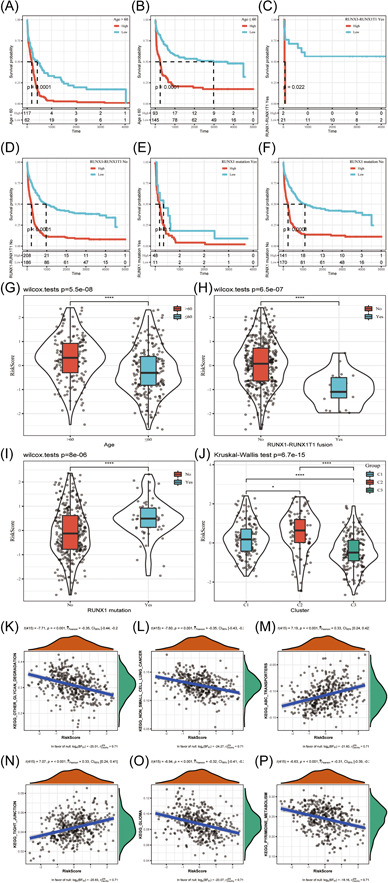
The classified performance of m^5^C‐score on prognosis of AML cases with different clinical features in training set. (A) Comparison of survival of patients over 60 years old. (B) Patients younger than 60 years old. (C) Patients with runx1–runx1t1 fusion (+). (D) Patients with runx1–runx1t1 fusion (−). (E) Patients with Runx1 mutation (+). (F) Patients with Runx1 mutation (−). Relationship of m^5^C‐score with clinical features of samples from training set. (G–J) Comparison of risk score between age (G), runx1–runx1t1 fusion (H). Runx1 mutation (I) grouped samples and our molecular subtype samples (J). GSVA conducted to examine biological behaviors between patients with high and low m^5^C‐scores from training set. (K) KEGG_ OTHER_ GLYCAN_ The degree pathway was negatively correlated with the risk score of the sample; (L) KEGG_ NON_ SMALL_ CELL_ LUNG_ The cancel pathway was negatively correlated with the risk score of the sample; (M) KEGG_ ABC_ The transporters pathway was positively correlated with the sample risk score; (N) KEGG_ TIGHT_ There was a positive correlation between the junction pathway and the sample risk score; (O) KEGG_ Glioma pathway was negatively correlated with the risk score of the sample; (P) KEGG_ PYRIMIDINE_ Metabolism was negatively correlated with the risk score of the sample. AML, acute myeloid leukemia.

**Figure 10:**
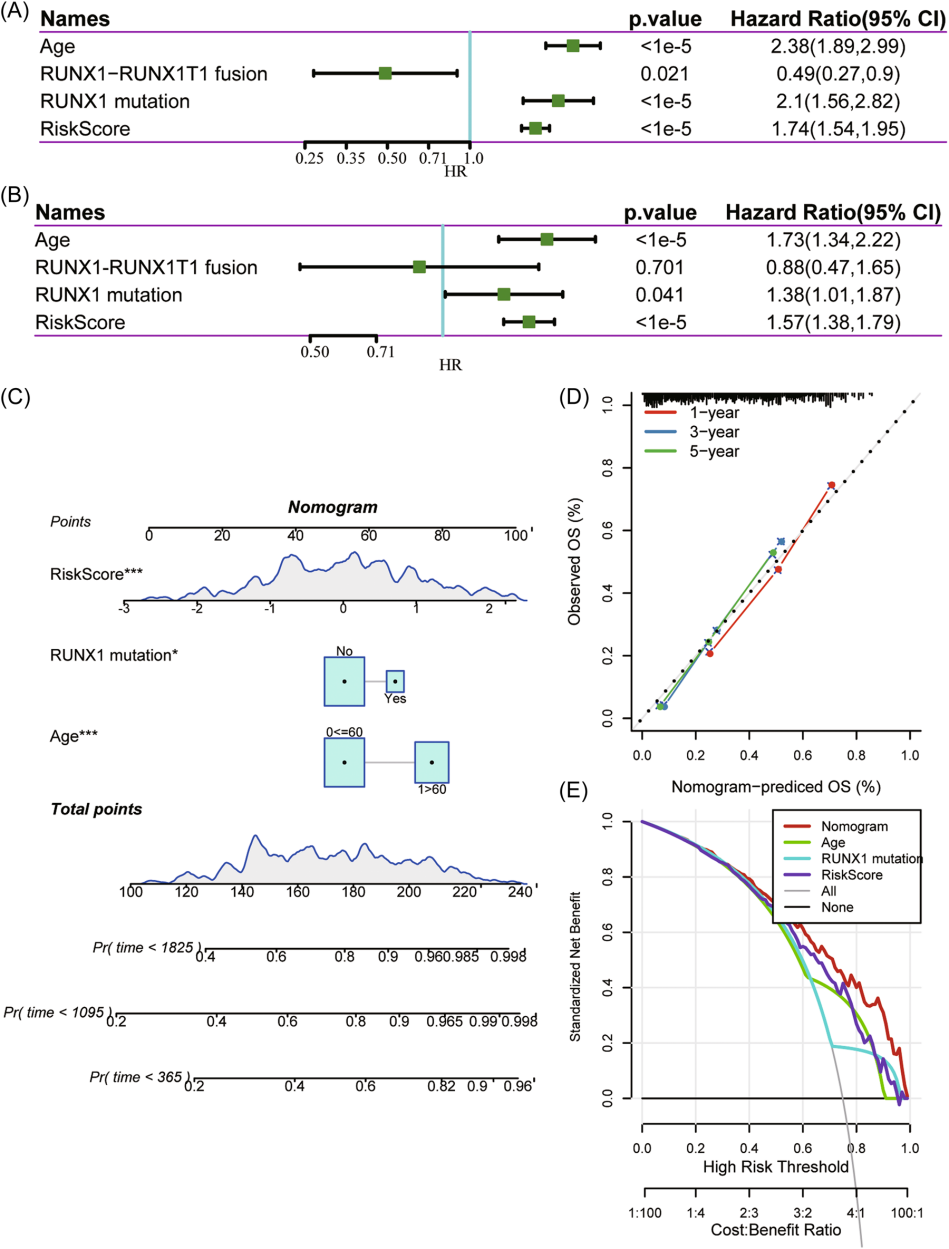
Assessment of the value of m^5^C‐score in predicting the prognosis of AML. (A) Univariate analysis suggested the m^5^C‐score was significantly related to OS (HR = 1.74, 95% CI = 1.54–1.94, *p* < 1e^−5^). (B) Multivariate analysis demonstrated m^5^C‐score served as the factor to independently predict OS (HR = 1.57, 95% CI = 1.38–1.79, *p* < 1e−5). Establishment and evaluation of nomogram model. (C) Nomogram predicting the probability of training set patient's mortality based on m^5^C‐score and clinical variables. (D) Calibration curves of the nomogram for 1, 3, and 5 years. (E) Decision curve analysis of the nomograms based on the m^5^C‐score. AML, acute myeloid leukemia; CI, confidence interval; HR, hazard ratio; m^5^C, 5‐methylcytosine; OS, overall survival.

First, we tested the ability of m^5^C‐score in classifying the prognosis of patients with diverse clinical features and gene mutations. We classified GEO‐AML samples (GSE37642) according to the specific clinical features, like age, RUNX1 mutation and RUNX1–RUNX1T1 fusion. According to Figure 9A–E, the constructed m^5^C‐score can classify cases into two subgroups with different OS, such as young or old cases, those with or without RUNX1–RUNX1T1 fusion, and those with or with no RUNX1 mutation according to median score. Second, this study assessed the value of m^5^C‐score in predicting the prognosis of AML. As suggested by univariate analysis, m^5^C‐score was significantly related to OS (HR = 1.74, 95% CI = 1.54–1.94, *p* < 1^e^ ^−5^) (Figure 10A). Moreover, as demonstrated by multivariate analysis, m^5^C‐score served as the factor to independently predict OS (HR = 1.57, 95%CI = 1.38–1.79, *p* < 1^e^ ^−5^) (Figure 10B). To improve prediction performance, we constructed a nomogram based on the m^5^C‐score, RUNX1 mutation, and age (Figure 10C), and it was easy to predict OS for individual patients through calculating the overall nomogram score (Figure 10D). For 1‐, 3‐, and 5‐year survivals, the calibration curves were consistent with the standard curve, indicating that the model had favorable prediction accuracy. Besides, we used DCA for assessing the model robustness (Figure 10E), which showed that the established m^5^C‐score and nomogram had remarkably increased profits compared with a limit curve, indicating their favorable robustness.

## 4 DISCUSSION

Epigenetic alterations have contributed greatly to human carcinogenesis. Conventional epigenetic studies have been predominantly focused on DNA methylation, histone modifications, and chromatin remodeling. Epitranscriptomics is an emerging field that encompasses the study of RNA modifications that do not affect the RNA sequence but affect functionality via a series of RNA binding proteins called writer, reader, and eraser. Several kinds of epi‐RNA modifications are known, such as 6‐methyladenosine, 5‐methylcytidine, and 1‐methyladenosine. AML is a type of blood cancer affecting specific subsets of blood‐forming hematopoietic stem/progenitor cells (HSPCs), which proliferate rapidly and acquire self‐renewal capacities with impaired terminal cell‐differentiation and apoptosis leading to abnormal accumulation of white blood cells, and thus, an alternative therapeutic approach is required urgently.^34^, ^35^, ^36^, ^37^

More and more studies suggest that the interaction between m^5^C modification with different m^5^C regulators has a key function in innate immunity, inflammatory, and anticancer effect. Many studies have focused on one single regulator or cell type in cancer TME, while research for the comprehensive identification of immune cell infiltration features in TME mediated by diverse m^5^C regulators is lacking at present. Therefore, it is important to identify different patterns of m^5^C modification within TIICs in TME so as to present anticancer immunity and guide efficient immunotherapy.

Using nine m^5^C regulators, this study identified three different patterns of m^5^C modification in AML, which showed various TME‐infiltrating features. In addition, diverse mRNA transcriptome data across the different patterns of m^5^C modification were remarkably related to biological pathways related to m^5^C as well as immune inflammatory responses. We deemed such DEGs as genes related to patterns of m^5^C modification. According to our results, m^5^C modification had an important function in the formation of diverse clinical features, as well as TME landscapes and prognosis in AML patients. Consequently, it is of great importance to comprehensively assess m^5^C modification patterns for revealing TME‐infiltrating features. Due to the heterogeneous m^5^C modification patterns, quantifying these patterns in individual AML patients is of great importance. Therefore, the present work built the m^5^C‐score system (involved five prognostic genes related to m^5^C‐ modification patterns). Then, we evaluated m^5^C‐scores to comprehensively assess patterns of m^5^C modification in individual AML samples, and the score was later used to classify AML prognosis in a more effective and stable manner.

Among the five genes, four (LAPTM4B, IGLL1, HOPX, and HIST1H2AE) were related to AML genesis, development, and malignant transformation. In addition, these genes were significantly associated with the diagnosis, prognosis, and survival of patients.^38^, ^39^, ^40^, ^41^ Thus, bioinformatics analysis in this study showed high reliability and accuracy, and it combined screening based on the GEO database as well as validation using TCGA database. But the association of such genes with TME‐infiltrating features in AML is not illustrated yet. Besides, the association of ITGA4 with AML has not been validated in fundamental or clinical studies at present. ITGA4 codes for the integrin α4 chain (or CD49d), which constitutes half of the α4β1 lymphocyte homing receptor and regulates homing, trafficking, differentiation, activation, and survival of lymphocytes, eosinophils, monocytes, macrophages, NK cells, basophils, and mast cells. ITGA4 has been demonstrated to be associated with prognosis and immune infiltrates in multiple types of cancer, such as multiple myeloma, breast cancer, and ovarian cancer.^42^, ^43^, ^44^ Although we validated the five genes in the m^5^C‐modification patterns with AML bone marrows by qRT‐PCR, we did not validate it with AML cell lines, making our results more conservative. In the meanwhile, our sample size is tiny, and we eagerly anticipate the validation of a larger sample size.

m^5^C‐score might be adopted clinically for the complete evaluation of patterns of m^5^C modification together with the relevant TME‐infiltrating features for individual patients, thus determining the AML immune phenotypes and guiding efficient clinical treatment. Moreover, the m^5^C‐score was also used to assess the clinicopathological characteristics of patients, including tumor inflammation stage, tumor mutation burden, molecular subtypes, and genetic variation. Besides, the m^5^C‐score served as the biomarker to independently predict the patient's prognosis. The adjuvant chemotherapy efficacy and anti‐PD‐1/PD‐L1 immunotherapy response may also be predicted by our constructed m^5^C‐score. This study proposed some new points regarding cancer immunotherapy. Specifically, patterns of m^5^C modification might be altered by targeting m^5^C phenotype‐related genes and m^5^C regulators or through abolishing the unfavorable TME‐infiltrating features in AML, thus facilitating the development of new immunotherapy or drug combinations.

## 5 CONCLUSION

To sum up, this study illustrated the mechanisms related to the regulation of patterns of m^5^C modification in TME from AML. Differences in patterns of m^5^C modification may be one of the indispensable parameters that might affect the diversity and complexity of TME. It is of great significance to comprehensively evaluate patterns of m^5^C modification across individual cancer samples (m^5^C‐score) so as to understand the TIIC features and guide efficient immunotherapy.

## AUTHOR CONTRIBUTIONS

**Qiang Wen**: Methodology; writing—original draft. **ShouJun Wang**: Methodology; writing—original draft. **Lili Hong**: Data curation; formal analysis; writing—review & editing. **Siyu Shen**: Data curation; software; writing—review & editing. **Yibo He**: Data curation; formal analysis; software; writing—review & editing. **Xiaofen Zhuang and Xianfu Sheng**: Data curation; formal analysis. **Shiliang Chen**: Data curation; software. **Ying Wang**: Writing—review & editing. **Haifeng Zhuang**: Funding acquisition; project administration; supervision.

## CONFLICT OF INTEREST STATEMENT

The authors declare no conflict of interest.

## ETHICS STATEMENT

This study was approved by the Ethics Committee of The First Affiliated Hospital of Zhejiang Chinese Medical University. All patients signed informed consent in accordance with the Declaration of Helsinki.

## Supporting information


**Fig.S1** Analysis of differences between different molecular subtypes (A) C1 and C3, (B) C1 and C2 (C) C2 and C3. GO analysis results between different molecular subtypes (D) C1 and C3, (E) C1 and C2 (F) C2 and C3.

**Table S1** 18 genes upon the p<0.01 threshold.

**Table S2** Differentially expressed genes among m5C molecular subtypes.

**Table S3** Univariate Cox analysis results.

## Data Availability

The data generated are included in the manuscript and supplementary data. Part of the data was obtained from TCGA and GEO databases.
