# Supplementary figures and images for: m5C regulator‐mediated methylation modification patterns and tumor microenvironment infiltration characteristics in acute myeloid leukemia
Source: Immun Inflamm Dis. 2024 Jan 22;12(1):e1150. doi: 10.1002/iid3.1150 (PMC10802208; doi:10.1002/iid3.1150)

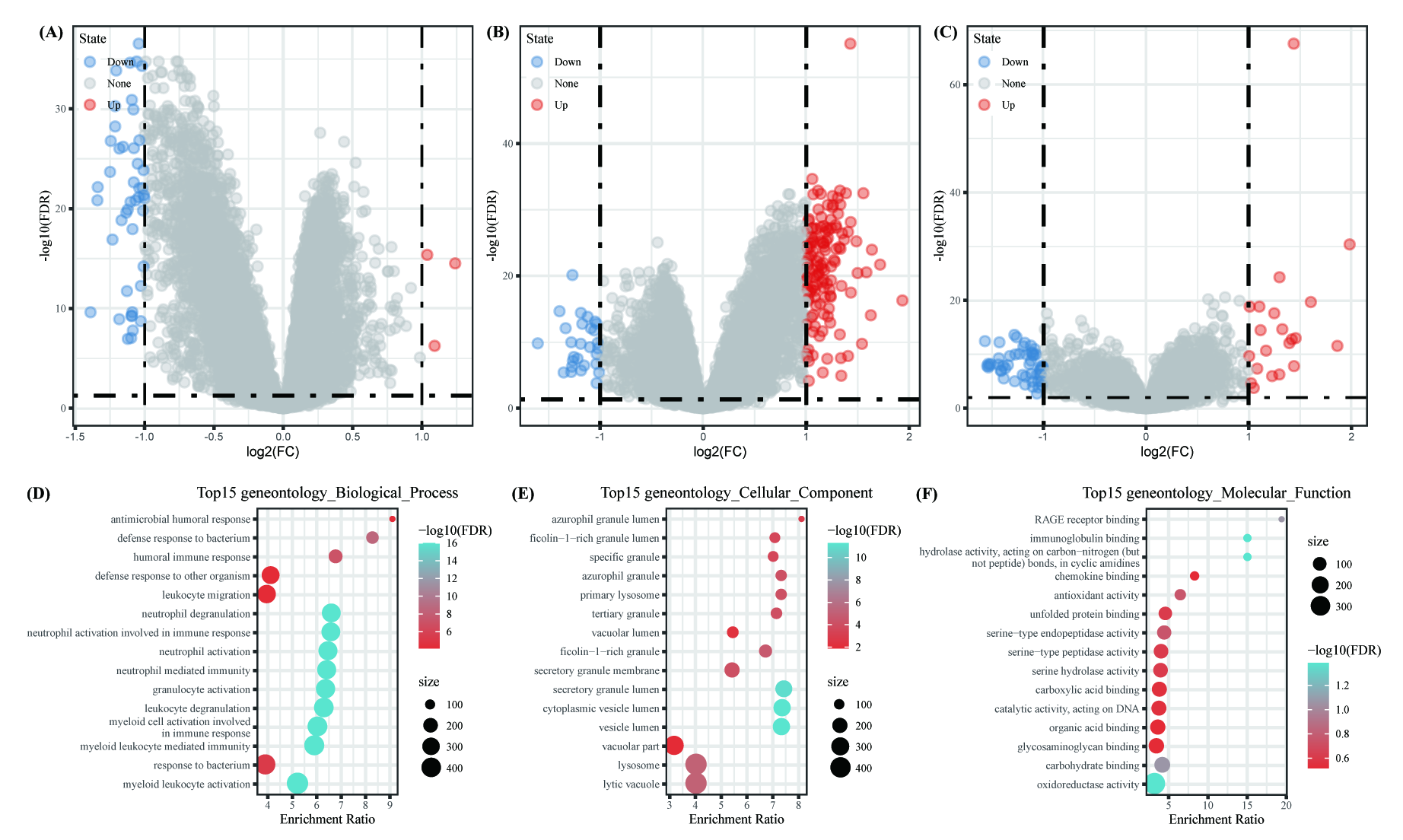

Supplement: Supplementary file 1 — Fig.S1 Analysis of differences between different molecular subtypes (A) C1 and C3, (B) C1 and C2 (C) C2 and C3. GO analysis results between different molecular subtypes (D) C1 and C3, (E) C1 and C2 (F) C2 and C3. [file IID3-12-e1150-s004.tif]
