# Supplementary material for: m5C regulator‐mediated methylation modification patterns and tumor microenvironment infiltration characteristics in acute myeloid leukemia
Source: Immun Inflamm Dis. 2024 Jan 22;12(1):e1150. doi: 10.1002/iid3.1150 (PMC10802208; doi:10.1002/iid3.1150)
Supplement: Supplementary file 4 — Table S3 Univariate Cox analysis results. [file IID3-12-e1150-s003.doc]

**Table S3 Univariate Cox analysis results**

p.value HR Low 95%CI High 95%CI

DNMT3B 0.000209353510430726 1.47173169724534 1.19978988050109 1.80531126647944

HADH 0.726913767622396 1.04301896245412 0.823442005709211 1.32114775357117

UNG 0.395551941995146 0.928909552588315 0.783591687221252 1.10117676203243

ACSL5 0.619881275212044 1.06242788533045 0.836334441356347 1.34964310413565

FAM216A 0.93396873839537 0.993019360078205 0.841374485148492 1.17199590300875

PMS1 0.534529333166353 1.05090951364108 0.898491171456945 1.22918381498449

NARS2 0.526799640411851 1.07087701946515 0.866247290583088 1.32384551534544

CEP83 0.722666507098658 1.04264349954738 0.827901067726515 1.31308620018294

C21orf59 0.744531168248991 0.967458667928037 0.792893422742724 1.18045660022179

ACYP1 0.946653606598519 0.993777691477067 0.827720882350984 1.1931487064485

HIBCH 0.357039237260715 0.920347502267341 0.771329888767958 1.09815467708986

THYN1 0.077970669448704 0.844036572357056 0.698998467352092 1.01916923820293

PFAS 0.315775058015105 0.907573250709442 0.750918515481065 1.09690890345888

DFFB 0.270732503765767 0.902501472209665 0.751908398081924 1.08325549949752

C14orf159 0.121940014340622 0.856467078090377 0.703787017877064 1.0422696600249

FKBP3 0.947844326947503 1.00627501409918 0.834292022441246 1.21371099898252

CDK4 0.252582212950686 0.896293218395023 0.743005666828767 1.0812051229295

RUVBL2 0.611108894551618 0.942614748937434 0.750596097402669 1.18375590812287

PIBF1 0.639070572811422 1.04499043944573 0.8694431526339 1.25598207913288

CCT7 0.180355216990668 0.857721125933448 0.685219251392842 1.07364982577053

POP5 0.66465906394754 0.963752915400445 0.815584274352967 1.13883961615092

ALG8 0.177822660851386 0.88585144495688 0.742669753005468 1.05663759612735

C11orf73 0.618588398249306 0.954248142659639 0.793580862405207 1.14744389753746

ATIC 0.203594858078834 0.878594764292964 0.719668791182424 1.07261669437509

QDPR 0.79511818692493 1.02830106765645 0.832985814386272 1.26941307700717

MTHFD1 0.672144423772886 0.957721589119353 0.784068894789826 1.16983424334308

CLNS1A 0.115405492687135 0.870668347174476 0.732779893496945 1.03450350848729

DPAGT1 0.942410874639771 0.992988578181597 0.820423268539326 1.20185074486566

DROSHA 0.499908857974344 1.07819473508243 0.866371836111467 1.34180710672351

RPP40 0.914723680576899 0.988688638443824 0.802846809375688 1.21754886781954

MAP7 0.00630583630348799 1.28238137385523 1.07277508317465 1.53294200602073

PRMT5 0.281104980971069 0.886493580876827 0.712078669021622 1.10362928019679

ACADM 0.90712266968452 1.0097504548389 0.857867804603104 1.1885234246774

TM7SF3 0.0998871787030679 0.853264391135394 0.706304546889937 1.03080197400047

MSH2 0.802821806641786 0.98062101325908 0.840990119894547 1.14343504031396

ICE2 0.998933570234305 1.00013791826366 0.817018916752324 1.22429950523655

GMDS 0.265978454048172 0.88784092337764 0.719951625114598 1.09488120830145

FASTKD1 0.872267996224719 1.01422588347984 0.853787741241121 1.20481249967955

PUS7 0.382637031030066 0.930972897730496 0.792869470169294 1.09313142820805

TIMM13 0.712969725991312 1.04015823611152 0.843327172278798 1.28292932057092

TTC37 0.652584098438545 0.959581309937879 0.80180690343615 1.14840154959507

DEPTOR 0.244174101746584 0.924556725554587 0.810218714239707 1.05503010945674

PIGF 0.615896365633615 0.953659363740612 0.792293438803594 1.14789058890036

PHKB 0.338886163636253 0.904998051172999 0.737568076643089 1.11043508872369

TMEM165 0.698576955537016 0.960810731113982 0.784805579576221 1.17628784127945

CRYZ 0.85707818588189 0.983940903952816 0.824994565015853 1.17351040058413

FANCL 0.334085903214742 1.091262947477 0.914041593301688 1.30284532920937

BANF1 0.358484161676709 0.90668836627407 0.735622229137672 1.11753527962365

TRMT13 0.6663536542628 1.04518060845871 0.854970290682928 1.27770814518657

FOCAD 0.340241633869564 0.916811982895686 0.76693914884613 1.09597249435725

ARHGAP22 0.0259819380750962 1.18482093904601 1.02050817605862 1.37558981940114

NME7 0.407521026003653 0.91135875723258 0.731644713128935 1.13521599962432

HSD17B4 0.0808609084158112 0.868164097868848 0.740744170005528 1.01750230558386

PRKACB 0.754078035193052 1.0269215097032 0.869669111604067 1.21260807474924

CCDC53 0.249414091115381 0.880926337031652 0.709976125400733 1.09303846074822

ANGPT1 0.156073191737377 1.09732568797714 0.96516032954657 1.24758926432481

NIT2 0.0632497045946145 0.84872098744257 0.713834682928436 1.00909542748808

IARS 0.93108984243753 1.00912860143381 0.821290981692017 1.23992659962463

SPG11 0.167162183305285 0.871178334434071 0.71635496580574 1.05946315250802

TMEM135 0.0632473705592335 1.19983371292946 0.989995419998642 1.45414908958273

MCTS1 0.468904161023787 0.926038154122612 0.752179801418283 1.14008201399965

DNAJB6 0.234868884644154 0.906636888217423 0.77126141418533 1.06577410973532

NUP43 0.639700190801302 0.951909368590635 0.774421665763343 1.17007501994079

SAC3D1 0.0520306418090151 0.847631381870197 0.717435355776383 1.00145463106382

SLC38A10 0.17257491735641 0.893092200510533 0.759166107609957 1.05064447769386

SKAP2 0.778596491260891 0.974148365077614 0.811572975036223 1.16929107593933

NME1 0.464067053014481 0.936228069679622 0.78482372968155 1.11684059146847

DENR 0.320951828659945 1.1291120370255 0.888369548883343 1.43509420573722

KIAA0125 1.10946179734571e-05 1.29074394610605 1.15187880422348 1.44635002250304

FAIM 0.792303637912788 0.974161115470553 0.801690485608222 1.18373598780439

PRDX1 0.271536765914514 1.11783768346368 0.916518821514649 1.36337744216362

C1QBP 0.148525708269999 0.881270719811955 0.742411178175098 1.04610235463711

GTF2H5 0.998185329188605 0.9997808896228 0.827735231466432 1.20758642287596

CCDC91 0.783680902566377 0.975314819136572 0.815914555763443 1.16585614229831

C11orf21 0.0370436987643558 1.1903230854224 1.01052530310478 1.40211140021557

PLAGL1 0.630945262778213 0.960442200679447 0.814621515585861 1.13236540307012

ATP8B4 0.378884691438479 0.947577031385764 0.840479023263029 1.06832199919029

LSM7 0.24552233756265 1.13304893277607 0.917679329567619 1.39896349705282

GUCY1A3 0.803394649974222 1.01661353513593 0.892934386824101 1.1574233169555

PIEZO1 0.0343673263912591 0.833065635446475 0.703393650518343 0.986642902520403

CCR7 0.945423189909252 1.00569517529333 0.85477361419554 1.18326392954955

TTC27 0.500889237460334 0.939453353843342 0.783233038081059 1.12683270640604

SEH1L 0.906091186553017 1.01061741718922 0.847972621081527 1.20445818477436

MKKS 0.714637935990987 0.965646369893967 0.800638353619845 1.16466180701124

DMXL1 0.636105403269517 0.960802025712863 0.814136532796171 1.13388909037578

IMPDH2 0.0985433796228672 0.844994637910134 0.691942214455527 1.03190110847439

FAH 0.338750483562085 0.924398736071352 0.786885876900794 1.08594276290213

PRMT1 0.236720645179604 0.901856656154828 0.760019145708427 1.07016439367805

CBX1 0.821169539111364 1.01884735123189 0.8665552910603 1.19790385659305

ADA 0.702429946740799 0.968078125601022 0.819648404881652 1.14338691039423

ANXA4 0.977829381916232 1.00206133043243 0.866606481420144 1.15868843757378

TCFL5 0.574298141686577 1.05249996957527 0.880412309293036 1.25822432769648

SSBP2 0.498327212331091 0.949191436696533 0.816212937245162 1.10383497049056

ITGA4 0.00454908988775631 0.784581125370145 0.663523559994628 0.927725222435307

CLIP2 0.630120135170174 0.963569557893806 0.828488281567685 1.12067522686356

MLC1 0.688966899318738 1.03587323507259 0.871673482828034 1.23100378786152

IQGAP2 0.914083415019283 1.00991403644899 0.844213053620211 1.20813858141967

DERA 0.164349202696513 0.890291982547074 0.755790106829837 1.0487300733695

ATP10D 0.809826009260558 0.979992523844801 0.831260343636437 1.15533641673606

FLT3 0.823786435749577 1.01351617847948 0.900554809011232 1.14064689207255

SMYD3 0.0767956543042221 0.87842322780658 0.760941361265763 1.01404313975862

GALNT7 0.472478167001542 0.946927047888756 0.816031970587308 1.09881826489956

PPA2 0.573839186959056 0.949815903712207 0.793804867029615 1.13648868684875

DYNLL1 0.461542617419588 0.94303388829408 0.806718678172005 1.10238294777828

FAR2 0.196693822416772 0.89716525273275 0.760897473499238 1.05783698690626

SUCLA2 0.923463715333225 1.0082254777203 0.853055739988391 1.1916203904075

SPINK2 0.0236004074905468 1.09801912092339 1.01262099978928 1.19061918542501

PTPLA 0.832318544407377 0.98324579119871 0.840882820333372 1.14971106857278

PYCARD 0.203621747658559 0.911036894417433 0.78913728717466 1.05176657658816

OPN3 0.245398443357556 0.925394906469739 0.811915589221531 1.05473493093194

LGALS3 0.793498544427805 0.982596958553568 0.861563983569213 1.12063271140809

RORA 0.365149275996989 1.06728415324531 0.926988031357966 1.22881356094735

LY75 0.126258353915146 0.896463833027349 0.779269017544017 1.03128365921553

VAMP8 0.0529903450134343 0.873902543512585 0.762378766544043 1.00174045903683

IGLL1 0.00146987193155637 0.846039885352693 0.763214863644689 0.937853180937037

WASF1 0.783980039833331 1.01765580144798 0.897960866558661 1.15330563812837

ITK 0.700419719335231 0.969252646469745 0.826696475511808 1.13639131230962

HIST1H2BD 0.0280286178151374 1.20683464907488 1.02048176213774 1.42721793200562

KLRB1 0.69700058844517 0.967014964383225 0.816787410649417 1.14487310792118

MMRN1 0.00620985499125526 1.17872888246905 1.04776749356467 1.32605925159958

BCL11B 0.646413471406698 0.96276340778681 0.818669558892491 1.13221918331398

RASGRP1 0.431482863504152 1.05529055581823 0.922869562386334 1.20671241374517

HIST1H2BH 0.0543246551278984 1.18938714975518 0.996785148538906 1.41920432309443

FTO 0.272598458891983 0.916962530211167 0.785403391218362 1.07055850689279

AQP9 0.436343364926539 1.04901506279579 0.92993682965711 1.18334124090796

FOXO1 0.0103217783334247 1.21447919944838 1.04689351422206 1.4088918365196

KRT1 0.985952821974702 1.00141370672964 0.855686479861844 1.17195893078491

TFEC 0.996461385265113 1.00027732454412 0.884914942996097 1.13067898097586

KDELC1 0.00257617105305367 1.21485282697688 1.07044307623675 1.37874439470643

LAPTM4B 2.63984447710082e-06 1.30052473574441 1.16546546404358 1.45123527076889

ATP1B1 0.525263615328307 1.04235297717199 0.917134816505992 1.18466741144835

CLEC7A 0.920944008806202 1.0051880862912 0.907538778475478 1.11334425898481

SLC25A37 0.840937945883635 0.98615620083433 0.860636751437989 1.12998201717403

ALAS2 0.87370056949129 1.00689727523615 0.925077804580772 1.0959533542559

PPP1R16B 0.0228847040721774 1.15613562239099 1.02031374044225 1.31003780933307

SEPP1 0.0210351875204032 1.10803794390517 1.01556568330629 1.20893025957367

CTSL 0.239770808929996 1.07123255896564 0.955123999266912 1.20145572330801

CCR1 0.710154836115512 1.0228171983774 0.908084181663245 1.15204629969488

FOSB 0.377033372452916 0.941020533995229 0.822285949618472 1.07689988599651

TMEM158 0.0383982976959421 1.12511038829345 1.00631726681257 1.25792672708021

ATF3 0.859434748990075 0.988509415445078 0.869824280892539 1.12338881069278

HIST1H2AE 0.00526431867790522 1.16569909689867 1.0466860034065 1.29824453569448

CYTL1 0.149631234227981 0.942071734761333 0.868621307848661 1.02173311363305

EPB42 0.352496106240514 1.0418709199626 0.955567893941549 1.13596848611796

AKR1C3 0.170457352345452 1.08498258794176 0.965546225971757 1.21919301683566

HOPX 5.74396540515046e-05 1.19353376838125 1.09497062241543 1.30096901880706

SELENBP1 0.386739193171979 1.04109325555304 0.950354575473715 1.14049555263913

MEST 0.435250330736288 0.963213250198077 0.876663958129898 1.05830718458677

G0S2 0.419107115871753 0.963122604984592 0.879218060922909 1.05503423264373

WBP5 8.83679254426642e-05 1.26044682809729 1.12271496489168 1.41507529171823

SLC4A1 0.405839004285481 1.04256543741638 0.944969470320817 1.15024106644017

AHSP 0.658172871522788 1.02094024913564 0.931385665919379 1.1191056835476

C5AR1 0.741314025646778 0.985763064328012 0.905312746022173 1.07336257361117

S100P 0.314347184108769 0.957548536474714 0.879958209205725 1.04198039192394

SERPINB2 0.840469206417548 1.00811610263856 0.931813118247938 1.09066727705024

MAFB 0.37352250969838 1.03236915212815 0.962418221212968 1.10740428929385

BASP1 0.677335121923649 1.02093440282922 0.926009368873487 1.12559018290307

FCER1A 0.646213927327888 1.02079502962148 0.934916089325684 1.11456258416891

PROM1 0.621156432592374 1.01807656852099 0.948253564466323 1.09304086819311

CPA3 0.441954104713178 0.969113081307535 0.894627335006683 1.04980043377992

LTF 0.99076816387115 0.999550610590584 0.92627035314442 1.07862830731908

TRAF5 0.78857480228002 1.02279729840819 0.867438508090475 1.20598094720736

HK3 0.265724340782371 0.925315504324422 0.807095914071702 1.06085134073308

NGFRAP1 0.000131995834465295 1.27280239860249 1.12473003647858 1.44036870479639

MNDA 0.377912622723904 0.956943652145364 0.867761359126394 1.05529146204806

NCF1 0.229255857399329 0.93778122216508 0.844541912314172 1.04131435968127

CECR1 0.00517475960997136 0.833861698163815 0.734143281522288 0.947124831303835

SERPINB10 0.275344573314894 0.925914323983328 0.806318858929238 1.063248522422

SCPEP1 0.277127751933318 0.933512744315323 0.824582348080334 1.05683325119425

S100A11 0.109725294468783 0.901992783947342 0.794892201267815 1.02352366898988

ALOX5AP 0.743716188179106 1.01997730615907 0.905922162064096 1.14839193547173

FCER1G 0.098991715625957 0.900340405214919 0.794768493928656 1.01993580703682

CDA 0.564832271493209 0.966563228818523 0.86088480897672 1.0852142650938

NCF2 0.899518550725452 0.993024588788381 0.89078515413627 1.10699850503737

FBP1 0.290008191934408 0.937586578713676 0.832083755732781 1.05646647531276

FCGR2A 0.383782714197677 0.94685652706051 0.837364499249751 1.07066550306391

CKAP4 0.931640741898864 0.994265919211451 0.871845518875359 1.13387600980113

CEBPD 0.821438677387726 0.986362298793437 0.875484282378179 1.11128275408696

MS4A6A 0.230080088916302 0.935810443495286 0.839720878174713 1.04289557270319

MLLT11 0.346039607175061 0.932280247004966 0.805756360352059 1.07867154604387

CACNA2D3 0.310188877168184 0.929135987485949 0.806175925967428 1.0708502393017

ELANE 0.00323192342994202 0.894152349178894 0.82998891697772 0.963276023556344

CSTA 0.0838067090754174 0.910667186391473 0.819013198947926 1.01257797241298

S100A9 0.778217310094238 1.01359614302547 0.92267880234407 1.11347214062581

CD14 0.837572049222236 1.00836906778589 0.931138046820838 1.09200583129328

AZU1 0.00524887560482114 0.891829469363618 0.822947024141247 0.966477524182505

TREM1 0.566977726076616 0.966101739294115 0.858517409148179 1.08716790215493

PLBD1 0.591435182760793 0.978618023448091 0.904351882710241 1.05898296241431

RBM47 0.176443679966893 1.0804473999188 0.965787330570702 1.20872012609802

S100A12 0.99816719236205 0.999907525987357 0.924041223015483 1.08200265921405

HP 0.91539653581308 0.993557281029573 0.88186805834141 1.11939202395366

CD1D 0.527570704213533 1.02773748836626 0.944096986162057 1.11878796402818

KRT18 0.622164580697888 1.02825925077489 0.920378771764774 1.14878473867535

FGR 0.989352288475242 1.00076292078508 0.894722928371804 1.11937046862188

P2RY13 0.682174990052686 0.97718472331715 0.874988363184431 1.09131735193505

FCN1 0.664525750525045 1.0189110335905 0.936191795282059 1.10893910799514

CLEC5A 0.00848794851261707 0.858378980907672 0.766112216366487 0.961757898025244

CTSH 0.941076966896983 0.995837698046572 0.89157220955165 1.11229658150672

LY86 0.329949212816059 0.948038210059599 0.851536398710042 1.05547625338685

RNASE3 0.0525850043053732 0.905076836333905 0.818252016130675 1.00111464868957

FGL2 0.262162887410648 0.948846252007518 0.865636084040414 1.04005508382513

MS4A3 0.265712081902931 0.951068790733438 0.870649620624552 1.03891602692976

VCAN 0.886815205896501 0.994693232756497 0.924418904365087 1.07030981584169

DEFA4 0.980236547085191 1.00098818339197 0.925743756280614 1.08234847547449

RNASE6 0.432931606998253 0.961889088342192 0.872867286622047 1.05999002649345

DEFB1 0.310688492091666 1.05114852315304 0.954509710731464 1.15757147916294

COL4A5 0.820969929761593 1.01122169146674 0.91806177294611 1.11383497214068

MPO 0.00188910165671474 0.887466247316699 0.82308807049636 0.956879790095155

LCN2 0.797126313081041 0.990025177332105 0.917174240105153 1.06866264761109

PRTN3 0.526668130542731 0.974282854147459 0.898785491135621 1.05612194372026

HOXA5 0.00907464606653765 1.11600702049119 1.0276876935834 1.21191649716349

CEACAM8 0.722841605568451 0.987736382520424 0.922626865475152 1.05744065977538

TECR 0.104716236671416 0.844219565317404 0.68803286557868 1.03586138122236

CKS1B 0.210792576873738 0.879188791654488 0.718624261568323 1.07562877112435

IMPA2 0.118139020807216 0.886376619885866 0.761934192478994 1.03114352923852

PYGL 0.0143148355823736 0.83767348728363 0.726971244573196 0.965233324613663

PSMB10 0.143401525414792 0.885218989812996 0.751842484076471 1.04225642541083

PLAC8 0.940455479220751 0.993294773070549 0.83255427042974 1.18506929968662

TOP2A 0.808149092410759 1.01552438337797 0.896781753788974 1.14998969245073

PFN1 0.0840304523846412 0.869820358732321 0.742538359464104 1.0189203652876

SLC35A1 0.249811620645226 0.906410815582282 0.766733631284264 1.07153323277134

CDC20 0.885766654344714 1.01026462858825 0.878875441351092 1.16129609698449

PPIB 0.520098342308621 0.954947909330205 0.829799234859468 1.09897125861844

ECRP 0.417729624532171 0.946805467979311 0.829554033095658 1.08062954121296

SCD5 0.720651037919518 1.02729476628284 0.886326481842631 1.19068374741339

RRM2 0.912159816263997 0.992173502732429 0.862898775826463 1.14081545495472

HCK 0.18917382013797 0.913987607999365 0.799163523983913 1.04530965504028

DPYD 0.780461401710696 0.983609816738918 0.875694539076715 1.1048239179443

MS4A4A 0.414979455659755 1.05152902611671 0.93186829989873 1.18655532427288

DPYSL2 0.343573524507522 1.04531106657682 0.953716452633591 1.14570239707059
